# Musculoskeletal Disorders and Working Posture among Dental and Oral Health Students

**DOI:** 10.3390/healthcare4010013

**Published:** 2016-01-23

**Authors:** Andrew Ng, Melanie J. Hayes, Anu Polster

**Affiliations:** Melbourne Dental School, The University of Melbourne, 720 Swanston Street, Melbourne 3010 VIC, Australia; ng.andrew4@gmail.com (A.N.); apolster@unimelb.edu.au (A.P.)

**Keywords:** musculoskeletal disorders, dental, students, risk

## Abstract

The prevalence of musculoskeletal disorders (MSD) in the dental professions has been well established, and can have detrimental effects on the industry, including lower productivity and early retirement. There is increasing evidence that these problems commence during undergraduate training; however, there are still very few studies that investigate the prevalence of MSD or postural risk in these student groups. Thus, the aim of this study was to determine the prevalence of MSD and conduct postural assessments of students studying oral health and dentistry. A previously validated self-reporting questionnaire measuring MSD prevalence, derived from the Standardised Nordic Questionnaire, was distributed to students. Posture assessments were also conducted using a validated Posture Assessment Instrument. MSD was highly prevalent in all student groups, with 85% reporting MSD in at least one body region. The neck and lower back were the most commonly reported. The final year dental students had the highest percentage with poor posture (68%), while the majority of students from other cohorts had acceptable posture. This study supports the increasing evidence that MSD could be developing in students, before the beginning of a professional career. The prevalence of poor posture further highlights the need to place further emphasis on ergonomic education.

## 1. Introduction

Musculoskeletal disorders (MSD) are defined as muscular pain or injuries to the human support system that can occur after a single event or cumulative trauma, negatively impacting daily activities [1]. MSD can range from pain in the upper limbs, such as the forearm and wrist, to postural muscles such as the upper and lower back, neck and shoulders as well as lower extremities such as hips, thighs, knees and ankles. Left untreated, MSD can evolve into more severe degenerative and inflammatory conditions [1].

Of all work-related complaints, MSD may be the most ubiquitous symptom in the modern workforce. A study in 2011 revealed that MSDs were the most frequent health complaint by European, United States and Asian Pacific workers [2]. MSD is common in occupations that involve repetitive movements and prolonged, static postures such as sitting or standing, both of which are prerequisites to dental clinicians [3,4,5,6]. Studies have indicated that MSDs are multifactorial and are not just limited to physical causes. Psychosocial factors such as stress have also been discovered to be significant contributors in developing MSD [7].

Prolonged static postures (PSP) are inherent in dentistry work. Awkward postures that involve forward bending and repeated rotation of the head, neck and trunk to one side are common occurrences during clinical work. As posture deviates more from neutral, the muscles that are responsible for the preferred side of rotating or bending become stronger and the matching antagonistic muscles become elongated and weakened, creating a muscle imbalance [8]. Muscles that are under stress from PSP are also susceptible to ischemia, due to the prolonged contraction and following fatigue [9]. Under normal conditions, damaged tissues under these conditions are repaired during periods of rest. However, in dentistry the rate of damage exceeds the rate of repair due to insufficient rest periods, potentially leading to necrosis of the muscle. The body, in an effort to protect the stressed area from further pain or injury, compensates by using another part of the muscle to maintain posture. This is known as muscle substitution [8]. This is a self-perpetuating cycle, where tighter muscles become tighter and the weaker muscles become weaker, and can result in the development of a whole range of MSDs.

It has been well established in previous studies that there is a strong association between MSD and the clinical burdens of dental practitioners [10,11,12,13]. A previous review in this area discovered that 64%–93% of dental professionals suffer from general work-related MSD [10], representing a significant proportion of the workforce. This can have overall detrimental effects on the industry’s work force, resulting in lower productivity, increased sick leave and early retirement from the profession [1]. There is also some evidence to suggest that MSD can develop in students during their education and training [14,15,16,17,18]. This may be attributed to the pressures of tertiary study and the physical burden of clinical training. With increasing numbers of students, and therefore professionals, in the dental health industry, it is crucial to determine the prevalence and address the aetiology of MSD involved with this profession.

Despite a widespread awareness of this occupational health burden, a recent literature review found that dental professionals continue to suffer from MSD at alarming rates [13]. Research suggests that symptoms may arise during the education and training of students [14,15]. Students are taught and are aware of the risks of MSD during the early phase of their training, but over time, less emphasis is given to this occupational health issue [16]. Research in this area has improved in recent years; however, there have only been a limited number of studies in Australia [19]. Studies also focus more on professionals in the industry rather than students. Dental and oral health students are a useful group to research as it is logical to hypothesise that symptoms may potentially arise during this educational phase of dental professionals. With an ever-increasing student population and, subsequently, registered professionals in the field of oral health and dentistry in Australia, research would be crucial in understanding potential occupational health problems as well as providing support for further research into MSD in the related dental professions.

Thus, the aim of this study was to determine the prevalence of musculoskeletal pain and postural deficits in dental and oral health students and to investigate any risk factors that may influence the rate of MSD in this population. This will be achieved by:

- Measuring MSD prevalence using a self-administered questionnaire
- Posture assessment

## 2. Methods

This study was carried out as descriptive and exploratory research, using a cross-sectional approach. The study examines the prevalence of MSD in students studying the Bachelor of Oral Health (BOH) and Doctor of Dental Surgery (DDS) degrees at the University of Melbourne, Australia. The study duration of the BOH and DDS, respectively, are three and four years to completion. In addition to assessing the prevalence of MSD, the study also conducted an assessment of posture as an influencing factor of MSDs.

A cross-sectional design was selected as this was identified as the most appropriate method to answer the research question. Cross-sectional studies measure exposures and outcomes at a single point in time, and, as such, are useful for yielding prevalence estimates. This study design is efficient in terms of cost and time, as it allows several factors to be studied; given that this project is required to be completed in less than 12 months, a cross-sectional approach was considered the most appropriate choice. Ethics approval was obtained from the University of Melbourne in August 2015.

The questionnaire was two pages long and included a plain-language statement explaining the study; consent was implied by completing the questionnaire. The questionnaire was an adapted version of an original tool by Smith and Leggat [20] that has been previously used among medical, dental, nursing and occupational therapy students, as well as dental hygienists and dentists [10,21], and takes less than five minutes to complete.

Students were also invited to participate in a postural assessment, and were given a consent form to complete and return should they wish to participate. The postural assessment used was the Branson’s Dental Operator Posture Assessment Instrument (Table 1) [22], and was conducted during a timetabled clinical or pre-clinical scaling session, with assessment done by the student researcher. Scores ranged from 10 to 194, with the lowest score being the most ideal. From the scores, participants’ postures were categorised as Acceptable, Compromised or Harmful. These categories can be interpreted as follows:

➢ Acceptable (10–40): Postures in this category will not put the operator at risk for musculoskeletal discomfort or cumulative trauma disorders.
➢ Compromised (41–80): Postures in this category, if held for more than five minutes repeatedly throughout the work day, will put the operator at risk for musculoskeletal discomfort or cumulative trauma disorders.
➢ Harmful (81–194): Posture in this category, if held for any length of time, will put the operator at risk for cumulative trauma disorders or injury.

**Table 1:** Branson’s Dental Operator Posture Assessment Instrument (PAI).

Acceptable	Compromised	Harmful	1 min	3 min	5 min	Total
HIPS						
Level on stool (1 POINT)	Hips not level on stool (2 POINTS)					
TRUNK						
Front to back ≤20° (1 POINT)	Front to back >20°, <45° (2 POINTS)	Front to back ≥45° (3 POINTS)				
Side to side ≤20° (1 POINT)	Side to side >20°, <45° (2 POINTS)	Side to side ≥45° (3 POINTS)				
Rotation between planes ≤20° (1 POINT)	Rotation between planes >20°, <45° (2 POINTS)	Rotation between planes ≥45° (3 POINTS)				
HEAD/NECK						
Front to back ≤20° (1 POINT)	Front to back >20°, <45° (2 POINTS)	Front to back ≥45° (3 POINTS)				
Side to side ≤20° (1 POINT)	Side to side >20°, <45° (2 POINTS)	Side to side ≥45° (3 POINTS)				
Rotation between planes ≤20° (1 POINT)	Rotation between planes >20°, <45° (2 POINTS)	Rotation between planes ≥45° (3 POINTS)				
SHOULDERS						
Relaxed (1 POINT)	Slumped forward (2 POINTS)					
Both shoulder level with trunk (1 POINT)	One or both shoulders elevated above line of trunk (2 POINTS)					
WRIST						
Flexion or extension ≤15° (either wrist) (1 POINT)	Flexion or extension ≥15° (either wrist) (2 POINTS)					
TOTAL						

Sourced from: Branson, *et al.* [22].

All data was de-identified by assigning each individual that completes the survey with a numerical code. This code was used to identify data extracted from participants within the populations during data analysis. Students were not advantaged or disadvantaged by the study, as they were not identifiable in the data and were not directly involved in the study.

### 2.1. Participants

Participants included in the study were all students in their first and final years of the BOH and DDS programs at the Parkville campus of the University of Melbourne, Victoria. No exclusion criteria were applied to the selection of students. The participants were recruited via convenience sampling as this was the most time-efficient and effective method to obtain an appropriate sample relevant to the research question. The recruitment strategy involves the student researcher approaching students during a timetabled lecture, tutorial or clinical session and inviting them to complete a questionnaire. The students who wish to participate will be given time to complete and return the questionnaire during the session.

### 2.2. Data Analysis

Data yielded from the project was entered into a spreadsheet and analysed using the STATA statistical software package. After collecting the data, tabular summaries of demographic characteristics, prevalence of MSD, posture grades and risk factors were created.

More complex analysis of data was completed using regression analysis. Regression analysis can be used to describe the relationship between reported MSD and variables, including age, gender, study habits and exercise. Logistic regression was deemed the most appropriate method of data analysis for the data obtained from the surveys as it allows the dependent variable to be defined and correlation predictions to be made. Similar studies also used this method, making it more comparable to previous studies, particularly when the same survey has been used. Results are expressed as Odds Ratios (OR) with 95% Confidence Intervals (95% CI). The Fischer’s Exact test was used to analyse the data obtained from the posture assessments, as it allows for an independent variable to have two or more levels to be analysed against an ordinal or categorical, dependent variable. The Wilcoxon-Mann-Whitney test was also used, with first year students of both degrees acting as a control for their senior counterparts, as it allows the analysis of an independent variable with two levels against an ordinal dependent variable. Results with *p*-values of less than 0.05 were taken as statistically significant.

## 3. Results

Across all four cohorts, a total of 136 students completed the MSD survey and 138 consented to having their posture assessed. A perfect response rate was not able to be attained, due to absences or discontinued enrolment in the specified course. Participant numbers differ slightly between test types, as some students either withheld permission for a postural assessment or did not adequately complete the survey. However, the highest possible rate of participation was still acquired in all cohorts except DDS4. This is attributed to the inconsistent schedule of students and the difficulty in coordinating a suitable time for the experiment to be conducted. There was some level of inequality between the numbers of participants of both courses, as the number of enrolments in the DDS was much higher than in the BOH. BOH was also predominantly made up of female students (~80% +). Data detailing the number of participants in each cohort and the number that participated in each test is presented in Table 2.

**Table 2:** Description of gender distribution and specific numbers of the cohorts that participated in the study.

Gender Distribution of Participants						
Cohort	Test Type					
	MSD Survey			Postural Assessment		
	Male	Female	Total	Male	Female	Total
BOH1	11.11% (2)	88.89% (16)	18	11.11% (2)	88.89% (16)	18
BOH3	18.75% (3)	81.25% (13)	16	16.67% (3)	83.33% (15)	18
DDS1	55.26% (42)	44.73% (34)	76	55.84% (43)	44.16% (34)	77
DDS4	42.31% (11)	57.69% (15)	26	44% (11)	56% (14)	25

Further demographic data is illustrated in Table 3. An overwhelming majority of the population were non-smokers, with only one out of the 136 participants who reported smoking irregularly (0.5 packets per week).

**Table 3:** Demographic data of MSD survey participants.

	BOH1		BOH3		DDS1		DDS4	
	n	%	n	%	n	%	n	%
Age								
Mean	19.8	-	21.75	-	24.13	-	27.27	-
Range	19–24	-	20–28	-	21–35	-	24–32	-
Dental Practitioner experience								
Yes	4	22.22%	10	62.50%	6	7.89%	12	46.15%
No	14	78.78%	8	37.50%	70	92.11%	14	53.85%
Regular Drinker								
Yes	5	27.78%	6	37.50%	22	28.95%	4	15.38%
No	13	72.22%	12	62.50%	54	71.05%	22	84.62%
Exercise regularly								
Yes	12	66.67%	11	68.75%	54	71.05%	18	69.23%
No	6	33.33%	7	31.25%	22	28.95%	8	30.77%
Loupes								
Yes	1	5.56%	1	6.25%	9	11.84%	19	73.08%
No	17	94.44%	17	93.75%	68	88.16%	7	26.92%

**Figure 1:**
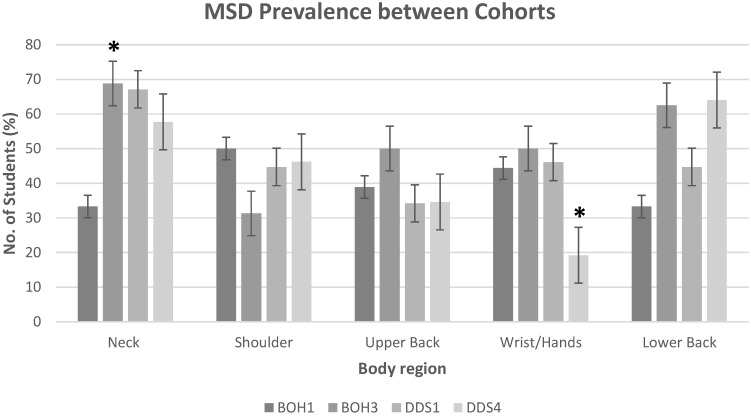
MSD prevalence of body regions between the cohorts studied. * *p*-value < 0.05.

The 12 month prevalence of MSDs by body region for all cohorts is presented in Table 4 and Table 5. Percentages calculated for other criteria such as pain lasting more than two days, seeking medical treatment, and effects on daily life were calculated relative to the number of participants that reported MSD in the particular region. That is, of the 33.3% of BOH1 students that reported neck pain, 50% of those reported that it lasted for more than two days. The consistently reported body regions with high rates of MSD across all cohorts were the neck and the lower back. BOH3, DDS1 and DDS4 presented high proportions of students with neck pain with 68.8%, 67.1% and 57.7%, respectively. However, BOH1 reported a lower rate at 33.3%. Lower back pain was also commonly reported among students; results also showed an increase in prevalence within the respective courses, from the first years and their senior counterparts. That is, BOH1 reported 33.3% and BOH3 62.5%; DDS1 reported 44.7% and DDS4 64%. Wrist/hand pain was also commonly reported among students (44.4%, 50% and 46.1% of BOH1, BOH3 and DDS1 students, respectively). Students studying the BOH had a significantly higher rate of wrist pain than students from the DDS (*p* < 0.03). A relatively lower percentage of DDS4 students reported wrist/hand pain (19.2%). Prevalence of MSD in the upper extremities was also common among students: 50% and 31.3% of BOH first and final year students, respectively, reported shoulder pain, while DDS first and final year students reported 44.7% and 46.2%, respectively. Upper back pain was also commonly reported, with all cohorts reporting approximately 35%, except for BOH3 which reported 50%. Elbows, forearms, hips/thighs, knees, calf/lower leg and ankles/feet pain had negligible numbers reported. Compared against their respective junior counterparts, BOH3 students showed a significant increase in the prevalence of neck pain, and DDS4 students demonstrated a significant decrease in the rate of wrist/hand pain (*p* < 0.05). Comparisons of MSD between body region and cohorts are illustrated in Figure 1. No other significant differences between groups were found.

**Table 4:** Prevalence of MSD by upper body region.

Reported MSD (% Students)				
Cohort	BOH1	BOH3	DDS1	DDS4
Neck				
Any symptoms	33.3	68.8	67.1	57.7
Persisted > 2 Days	50	36.4	41.2	40
Affected daily life	0	18.2	27.5	13.3
Needed treatment	0	0	9.8	6.7
Shoulders				
Any symptoms	50	31.3	44.7	46.2
Persisted > 2 Days	44.4	60	50	58.3
Affected daily life	44.4	20	23.5	0
Needed treatment	22.2	0	11.8	8.3
Upper Back				
Any symptoms	38.9	50	34.2	34.6
Persisted > 2 Days	28.6	62.5	46.2	22.2
Affected daily life	28.6	50	19.2	11.1
Needed treatment	42.9	12.5	7.7	11.1
Elbows				
Any symptoms	0	5.6	6.6	0
Persisted > 2 Days	0	0	100	0
Affected daily life	0	0	60	0
Needed treatment	0	0	0	0
Forearms				
Any symptoms	5.6	12.5	9.2	15.4
Persisted > 2 Days	0	50	71.4	25
Affected daily life	0	50	42.9	0
Needed treatment	0	50	28.6	0
Wrists/Hands				
Any symptoms	44.4	50	46.1	19.2
Persisted > 2 Days	25	25	60	40
Affected daily life	25	25	37.1	0
Needed treatment	0	12.5	8.6	0

**Table 5:** Prevalence of MSD by lower body region.

Reported MSD (% Students)				
Cohort	BOH1	BOH3	DDS1	DDS4
Lower Back				
Any symptoms	33.3	62.5	44.7	64
Persisted > 2 Days	66.7	50	50	62.5
Affected daily life	66.7	30	29.4	25
Needed treatment	33.3	20	5.9	18.8
Hips/Thighs				
Any symptoms	0	18.8	6.6	3.8
Persisted > 2 Days	0	100	60	0
Affected daily life	0	66.7	60	0
Needed treatment	0	33.3	40	0
Knees				
Any symptoms	11.1	6.25	14.5	7.7
Persisted > 2 Days	50	100	63.6	100
Affected daily life	50	100	45.5	0
Needed treatment	50	100	18.2	0
Calves/Lower Leg				
Any symptoms	5.6	0	3.9	11.5
Persisted > 2 Days	0	0	33.3	33.3
Affected daily life	0	0	33.3	0
Needed treatment	0	0	0	0
Ankles/Feet				
Any symptoms	5.6	5.3	6.25	15.4
Persisted > 2 Days	100	75	100	100
Affected daily life	100	25	0	25
Needed treatment	0	0	0	25

Statistical correlates of MSD were differentiated into two tables, with the predictors shown in Table 6 and the protective factors shown in Table 7.

**Table 6:** Statistical correlates and predictors of MSD.

Body Region	Predictor	OR *	(95% CI)	SEM	*p*-Value
Neck	Female	1.98	0.98–3.99	0.71	0.05
	DDS course	1.83	1.22–2.75	0.38	0.003
	DDS1 cohort	4.08	1.37–12.14	2.27	0.012
	BOH3 cohort	4.4	1.04–18.59	3.24	0.044
	No Loupes	1.52	1.03–2.25	0.3	0.034
	Computer type (Laptop)	1.64	1.14–2.35	0.3	0.008
	No previous practitioner experience	1.54	1.04–2.28	0.31	0.032
	Usage environment (Desk)	1.73	1.07–2.80	0.43	0.026
	Low preclinical hrs/week	1.88	1.16–3.05	0.47	0.011
	Low clinical hrs/week	1.7	1.08–2.67	0.39	0.021
Shoulder	Female	2.27	1.12–4.61	0.82	0.022
Upper Back	Usage environment (Table)	1.35	1.02–1.79	0.19	0.038
Wrists/Hands	Stress (high)	5.19	1.86–14.54	2.73	0.002
Lower Back	High clinic hrs/week	2.29	1.13–4.62	0.82	0.021

Note: *Odds ratio.

**Table 7:** Statistical correlates and predictors for non-reporting of MSD.

Body Region	Predictor	OR *	(95% CI)	SEM	*p*-Value
Shoulder	Male	0.49	0.28–0.84	0.14	0.01
	Low computer usage (hrs/week)	0.37	0.15–0.88	0.16	0.024
Upper Back	DDS course	0.52	0.35–0.79	0.11	0.002
	Male	0.53	0.31–0.90	0.15	0.02
	Low alcohol consumption	0.52	0.35–0.79	0.11	0.002
	No previous practitioner experience	0.58	0.39–0.86	0.12	0.007
	Low clinical hrs/week	0.5	0.32–0.79	0.12	0.003
	No Loupes	0.58	0.39–0.86	0.12	0.007
	Usage environment (Desk)	0.43	0.27–0.68	0.1	<0.001
Forearm	DDS course	0.12	0.06–0.23	0.04	<0.001
	BOH1 cohort	0.06	0.01–0.44	0.06	0.006
	Male	0.12	0.05–0.27	0.05	<0.001
	No previous practitioner experience	0.11	0.06–0.20	0.04	<0.001
	Low preclinical hrs/week	0.13	0.06–0.26	0.05	<0.001
	Low clinical hrs/week	0.11	0.05–0.23	0.04	<0.001
	Low computer usage (hrs/week)	0.18	0.06–0.53	0.09	0.002
Wrists/Hands	DDS course	0.65	0.43–0.96	0.13	0.031
	Low preclinical hrs/week	0.5	0.31–0.82	0.13	0.006
	Stress (low)	0.19	0.07–0.48	0.09	0.001
	Computer type (Laptop)	0.68	0.47–0.97	0.12	0.032
	Usage environment (Table)	0.38	0.15–0.97	0.18	0.044

Note: *Odds ratio.

DDS1 and BOH3 cohorts were both approximately four times more likely to develop neck pain (OR: 4.4 and 4.1; 95% CI: 1.37–12.14 and 1.04–18.59, respectively; *p* < 0.05). Not wearing loupes during clinical work (OR: 1.52, 95% CI: 1.03–2.25, *p* < 0.05), using a laptop as a primary computer (OR: 1.64, 95% CI: 1.14–2.35, *p* < 0.05) and having no prior experience in the field (OR: 1.54, 95% CI: 1.04–2.28, *p* < 0.05) also increased the risk of developing neck pain. Female students, compared to their male counterparts, were at greater risk of developing shoulder pain (OR: 2.27, 95% CI: 1.12–4.61, *p* < 0.05). Students who reported feeling high levels of stress in regards to the clinical requirements of their course was also an indicator of wrist/hand pain (OR: 5.19, 95% CI: 1.86–14.54, *p* < 0.05). Working many clinical hours also increased the risk of developing lower back pain (OR: 2.29, 95% CI: 1.13–4.62, *p* < 0.05).

Males were less likely to develop shoulder, upper back and forearm pain (OR: 0.49, 0.53, 0.12; 95% CI: 0.28–0.84, 0.31–0.90, 0.05–0.27; *p* < 0.05). Low computer usage (<10 h per week) was found to decrease the risk of developing MSD in the shoulders (OR: 0.37, 95% CI: 0.15–0.88, *p* < 0.05) and forearm (OR: 0.18, 95% CI: 0.06–0.53, *p* < 0.05). Having no prior experience as a dental practitioner also predicted a lower chance in reporting upper back (OR: 0.58, 95% CI: 0.39–0.86, *p* < 0.01) and forearm pain (OR: 0.11, 95% CI: 0.06–0.20, *p* < 0.001). In comparison to the BOH cohort, students from the DDS degree were less likely to develop upper back (OR: 0.52, 95% CI: 0.35–0.79, *p* = 0.002), forearm (OR: 0.12, 95% CI: 0.06–0.23, *p* < 0.001) and wrist/hand pain (OR: 0.65, 95% CI: 0.43–0.96, *p* < 0.05). Computer usage environment was also a protective factor, with the main environment as a desk, reducing the risk of upper back pain (OR: 0.43, 95% CI: 0.27–0.68, *p* < 0.001).

Posture assessment data showed that the majority of students were able to achieve an Acceptable grade of posture (BOH1 67%, BOH3 57%, DDS1 61% and DDS4 32%). The first year cohorts of both degrees demonstrated the highest percentage of students with Acceptable posture. The DDS4 cohort presented with the largest proportion of students with the Harmful grade of posture at 8% and Compromised at 60%, which was significantly higher than that of their DDS1 counterparts (*p* = 0.022). Most students from other cohorts that demonstrated poor posture fell into the Compromised category (33% of BOH1, 44% of BOH3, 36% of DDS1). Only two participants (2.6%) of the DDS1 cohort had Harmful grade posture. No other significant differences were found between overall score and cohort. 

Cut-off scores for specific body regions were determined based on the ideal score for that area (*i.e.*, a perfect score for the hips region would be 3—scores higher than 3 would signify that at some point in time during the assessment, the participant demonstrated poor posture). Refer to Table 1 for specific criteria used to assess posture.

It was found that 100% of all cohorts, except DDS1 with 95%, presented with poor neck positioning. Shoulder positioning was the next largest contributor to the scores, with approximately 65% of all cohorts achieving scores above 6. Only the DDS4 cohort had a higher percentage of students with poor shoulder positioning, at 92%. Within the BOH cohort, BOH3 showed a significantly larger amount of students with poor trunk posture (*p* < 0.03). Within the DDS cohort, the hips, trunk and shoulders were all significantly higher in the final years than in the first years (*p* < 0.05). Further details regarding the posture assessment results are illustrated in Table 8.

**Table 8:** Postural assessment results of students by cohort in percentage. Cut-off scores for specific body regions were calculated based on the ideal score for that area.

Postural Assessment Results										
Cohort	Body Area Score					Score			Average	SD
	Hips > 3	Trunk > 9	Neck > 9	Shoulder > 6	Wrist > 3	Acceptable	Compromised	Harmful		
BOH1	5.56% (1)	33.33% (6)	100% (18)	61.11% (11)	50% (9)	66.66% (12)	33.33% (6)	0	33.83	12.37
BOH3	5.56% (1)	72.22& (13) *	100% (18)	61.11% (11)	38.89% (7)	56.66% (10)	44.44% (8)	0	34.61	14.21
DDS1	6.49% (5)	46.75% (36)	94.81% (73)	68.83% (53)	18.18% (14)	61.04% (47)	36.36% (28)	2.6% (2)	35.79	19.47
DDS4	20% (5) *	88% (22) *	100% (25)	92% (23) *	76% (19)	32% (8) *	60% (15) *	8% (2) *	51.56 *	22.75

* *p*-value < 0.05.

## 4. Discussion

This study investigated the prevalence of MSD among students from the Bachelor of Oral Health and the Doctor of Dental Surgery courses. Posture was also investigated using Branson’s Dental Operator Posture Assessment Instrument [22]. It was found that 84.6% of all students surveyed suffered from MSD associated with the clinical requirements of their training. This finding is consistent with previous studies involving dental students, where the majority of the population suffer higher rates of MSD. Movahhed *et al.* reported that 82% of undergraduate students and 90% of postgraduate students reported pain in at least one body region [18]. This finding suggests that oral health professionals may have an increased risk of developing MSD during their education and training, well before the beginning of a professional career.

The body regions with the highest reported rate of MSD were the neck and the lower back, as detailed in Figure 1. In comparison to their respective junior counterparts, BOH3 displayed a significant increase in neck pain, and DDS4 students showed a significant decrease in wrist/hand pain. The increase in neck pain prevalence is similar to a previous longitudinal study of dental hygiene students that recorded increasing neck pain as students progressed through the course [15]. This may be the result of increased clinical hours students complete in their final year (100% of BOH3 students reported completing 16–20 clinical hours, compared to zero clinical hours in BOH1). The rate of wrist/hand pain was also significantly higher in BOH students, which further supports the premise that dental hygienists are more likely to suffer from wrist/hand MSD than dentists [10,21], as their role involves more repetitive, scaling tasks. It was interesting to see a significant decrease in wrist/hand pain in DDS4 students in comparison to DDS1, which may suggest that pain decreases over time for those in the DDS course. This may be the result of the difference in clinical procedures practiced in each year or the result of increased experience. However, no relevant protective factors were identified that may explain this finding, hence further research is needed to elucidate the deciding factor.

High levels of self-reported, clinic-related stress was found to be a predictor of wrist/hand pain. This is consistent with previous literature that report psychosocial factors as major contributors to MSD. Bernard *et al.* offers a plausible explanation for the mechanisms involved: that psychosocial demands may produce increased muscle tension or aggravate task-related biomechanical strain, or may affect awareness and reporting of musculoskeletal symptoms and perceptions of their cause [7]. This could indicate that people who reported higher levels of stress were more conscious of the musculoskeletal strain on their hands when conducting dental procedures, and hence were more likely to report wrist/hand pain. Low amounts of preclinical or clinical hours per week was also found to be a predictor of neck pain; however, this may be attributed to the lack of clinical experience, which was also discovered to be a predictor. Conversely, working high amounts of clinical hours per week predicts lower back pain. This finding supports previous assertions on the effects of prolonged, static posture and increased risk of MSD [8].

From a sample of 138 students, the posture assessment revealed that 61 presented with overall scores classified as Compromised or Harmful (~ 44.2%). No significant differences were found in the overall scores within the BOH cohorts. This could be the result of consistent emphasis placed on ergonomic posture throughout the course of the students’ training. It is noted that one of their clinical coordinators had obtained a Ph.D. exploring occupational health in the profession. Out of the four cohorts, DDS4 displayed the highest amount of students in the detrimental categories of posture and also had a significantly higher average overall score compared to the other cohorts (mean score = 51.56, *p* = 0.0015). This average score was also the only one to be classified as Compromised. It was also found to be significantly worse than that of their DDS1 counterparts, which suggests that in this degree, posture worsens over time. This places this cohort at the greatest risk of cumulative trauma disorder. It was mentioned by a clinic coordinator that DDS1 students were occasionally graded on their posture, providing incentive to maintain good form during clinical procedures. Perhaps this ergonomic education is not as heavily emphasised, or the increase in clinical and placement hours detracts from the time that could be allocated in the final years of study that result in this high prevalence of poor posture in DDS4. Ergonomic education including early instruction and monitoring of correct positioning requires further investigation in the prevention of MSD [13].

Only the prevalence of poor trunk posture was measured as significantly higher in BOH3 compared to their BOH1 counterparts (72.2%, *p* = 0.0028). Conversely, DDS4 presented with significantly higher prevalence of poor posture in the neck, trunk and shoulder regions (20%, 88%, and 92%, respectively) compared to DDS1. This further highlights the need for ergonomic eductaion in the later years of the DDS course.

Females were found to be more at risk of developing shoulder and upper back MSD. The BOH course was predominantly made up of females as well, which could also indicate a higher risk of neck pain. However, this gender imbalance in the BOH could also be an influencing factor in the MSD prevalence in this degree [15]. Females in the DDS course were also found to have a significantly higher prevalence of neck and shoulder pain than their male counterparts. This is further supported by the results of this study, which identified the female gender as a predictor of neck and shoulder pain. Being male was found to be a protective factor for upper back and shoulder pain. This conclusion is consistent with previous studies of MSD risk factors not only in dental professionals [23,24] but in students as well [15,16,17].

It was endeavoured to keep observational bias to a minimum; however, it was still a contributing factor towards the results of the posture assessments. Participants were aware of the observer during the procedure, as consent was first obtained before the assessment could proceed. It was found that BOH1 and DDS1 students had the highest percentage of students with Acceptable grade posture. This may be due to the difference in working environment compared to the other cohorts. Both BOH1 and DDS1 were assessed during a preclinical session, whereas BOH3 and DDS4 were assessed under a clinical setting while treating real patients. Hence, it could be concluded that participants who were not under the pressure of treating a live patient may have been able to shift a larger portion of their attention towards the observer and became much more conscious of their posture during the time of assessment.

Demographic data regarding height and weight were not gathered in the present study but may still be a noteworthy contributor to poor posture. It could be reasoned that those of greater height must strain to a greater extent than their shorter peers to be able to gain a clear view of the oral cavity. This could be likened to the study of working posture using a notebook personal computer (NPC), where the low height of the screen encouraged poor posture as it forced a steeper, downward view [25]. Weight has been shown to contribute to poor posture as found in a Czech study of school children, where those classified as having a low BMI had a higher occurrence of poor posture, while those with a high BMI had a lower occurrence [26].

Types of dental instruments and clinic environments were not measured in this study; however, the survey did record if participants wore loupes. Of the 136 students, 20.1% reported wearing loupes. This rate of usage is comparable to a Malaysian study of dental students that reported 19% [16]. Khan and Chew [16] found no statistical significance between dental loupes and the prevalence of discomfort in the neck and upper back. Conversely, the present study found that not wearing loupes was a predictor for neck pain but a protective factor against pain in the upper back. The effects of loupes on MSD and posture in the literature is mixed and quite limited. Hayes *et al.* described that overall levels of self-reported upper extremity MSD improved in dental hygienists but worsened in students. It was speculated that in students, the magnitude of meaningful clinical change as a result of wearing loupes is unclear, as they had less experience and reported comparatively lower levels of MSD symptoms than their professional counterparts [27]. However, further research is needed, as the long-term effects of loupe wear is still uncertain.

Inconsistency between the surveys used in similar previous studies of dental students present some difficulty in accurately comparing results. Although the information obtained was similar in regards to demographics and outcomes measured, differences in question structure and specificity can result in variation of data. A variation of the Standardised Nordic Questionnaire (SNQ) used in this study measures MSD using a series of binary, multiple choice questions, quantifying the subjective variable into categorical data. Demographic variables were also quantified into ordinal or categorical data using the multiple choice format, thus minimising potential difficulties that may arise during the analysis of data that is inherently subjective and difficult to quantify. This instrument is well established and has been used in previous studies of MSD in dentists, as described earlier. Movahhed *et al.* [18] used a questionnaire from Rising *et al.* [28] as a template for the instrument used in their study. Question structures were varied, including multiple choice, yes/no and open-ended. Not only does this lack of consistency in question structure impede the accurate comparison of results, but unquantified answers that would be acquired from open-ended questions could result in difficulties during data analysis. A similar study by Khan and Chew [16] used a pilot survey that was adapted from a questionnaire developed and used at the University of Connecticut, USA [29]. This questionnaire was much more detailed in regards to gathering data on independent variables. Demographic data such as height and weight, and other known influencing factors, for instance level of taught ergonomics and work environment, were incorporated into the analysis. This higher level of detail and specificity, which is absent from the SNQ, would allow a wider scope of comparison and a greater chance in identifying the risk factors of MSD.

## 5. Conclusions

The results of this study provide valuable insight into the epidemiological patterns of this occupational health issue. Dental and Oral Health students are reporting MSD at rates on par with professional oral health personnel, suggesting that MSD could be developed well before the beginning of a professional career. The high prevalence of poor posture in older dental students highlights the need for further emphasis to be placed on ergonomic education throughout the training of students. Future research can be done to elucidate the different clinical procedures that may promote poor posture and pose a higher risk in developing MSD.

## Abbreviations

The following abbreviations are used in this manuscript:

BOH	Bachelor of Oral Health (BOH1 first years; BOH3 final years)
DDS	Doctor of Dental Surgery (DDS1 first years; DDS4 final years)
MSD	Musculoskeletal disorders
PAI	Posture Assessment Instrument
PSP	Prolonged Static Posture
SNQ	Standardised Nordic Questionnaire
